# Association of Frailty Factors, Especially Handgrip Strength, With One-Year Clinical Prognosis Following Transcatheter Aortic Valve Implantation: A Retrospective Cohort Analysis

**DOI:** 10.7759/cureus.111171

**Published:** 2026-06-19

**Authors:** Kenji Tsujimoto, Shinichi Watanabe

**Affiliations:** 1 Department of Rehabilitation Medicine, Gifu University of Health Science, Gifu, JPN; 2 Department of Rehabilitation Medicine, Ichinomiya Nishi Hospital, Aichi, JPN

**Keywords:** frailty, handgrip strength, prognosis, rehabilitation, transcatheter aortic valve implantation (tavi)

## Abstract

Introduction: Frailty is an important determinant of prognosis in older patients undergoing transcatheter aortic valve implantation (TAVI). However, because frailty is a multidimensional construct, the specific components that most strongly influence long-term outcomes remain unclear. This study aimed to identify the preoperative frailty components associated with one-year composite outcomes after TAVI.

Methods and results: This retrospective cohort study included 123 patients who underwent TAVI. Preoperative frailty was assessed using the Clinical Frailty Scale (CFS), and its components were evaluated across physical (handgrip strength and short physical performance battery (SPPB)) and nutritional (serum albumin) domains. Univariate and multivariate Cox proportional hazards analyses were performed using multivariate models adjusted for age and sex. During the one-year follow-up period, 32 events (all-cause death or rehospitalization) occurred. In the univariate analysis, preoperative frailty, defined as a Clinical Frailty Scale (CFS) score ≥ 5 (hazard ratio (HR): 2.11, p=0.032), and handgrip strength (HR: 0.95, p=0.034) were significantly associated with the outcomes. In the multivariate analysis, only handgrip strength remained an independent predictor (HR: 0.93, 95% confidence interval (CI): 0.88-0.98, p=0.006). Receiver operating characteristic (ROC) analysis identified an exploratory cutoff value of 13.3 kg (area under the curve=0.622), and Kaplan-Meier analysis showed significantly lower event-free survival in patients with low handgrip strength.

Conclusion: Preoperative handgrip strength was an independent predictor of the one-year post-TAVI outcomes. It may serve as a practical tool for risk stratification and individualized rehabilitation planning.

## Introduction

Frailty has been increasingly recognized in geriatric medicine as a condition affecting numerous diseases. Frailty is a multidimensional and potentially reversible condition characterized by a decline in physical function and deterioration of nutritional status [1]. Older adults with frailty often experience adverse outcomes, including prolonged hospitalization, increased rates of readmission, and higher mortality, and its impact on cardiovascular diseases has been widely reported [2].

Transcatheter aortic valve implantation (TAVI) for aortic stenosis is a minimally invasive procedure, and its indications have been expanded to include elderly patients and those with multiple comorbidities. Consequently, a substantial proportion of patients who undergo TAVI present with preoperative frailty [2]. Frailty before TAVI is associated with poor postoperative outcomes such as increased mortality and hospital readmission [3]. Furthermore, frailty is not a single pathological entity; it comprises multiple components, including physical function and nutritional status.

TAVI enables early mobilization and the initiation of ambulation, and rehabilitation interventions are often implemented in the early postoperative period. Lauck et al. reported that mobilization and exercise therapy can be safely initiated as early as four hours post-TAVI [4]. In Japan, comprehensive rehabilitation programs are routinely provided from the early postoperative phase through multidisciplinary collaboration involving physical, occupational, and speech-language-hearing therapists. However, despite the provision of such early rehabilitation, certain patients with frailty still experience adverse outcomes, including hospital readmission or death, within one year of discharge.

Although the association between frailty and prognosis has been extensively investigated, the specific components of frailty that most strongly influence long-term post-TAVI outcomes remain unclear. Clarifying this issue is critical for planning and implementing individualized rehabilitation interventions that are closely aligned with prognosis, particularly in the context of limited clinical resources.

Therefore, this retrospective cohort study aimed to determine the preoperative frailty components associated with one-year composite outcomes of all-cause death or rehospitalization after TAVI.

## Materials and methods

This retrospective cohort study included 123 consecutive patients who underwent transcatheter aortic valve implantation (TAVI) at Ichinomiya West Hospital between November 2019 and December 2024. The inclusion criteria were patients who underwent TAVI and received preoperative frailty assessments, including a short physical performance battery (SPPB) [5] and handgrip strength evaluations. Patients with missing baseline frailty assessment data or incomplete one-year follow-up data were excluded from the analysis. All participants underwent in-hospital rehabilitation according to the hospital’s standardized protocol, which comprised 50-m gait training on postoperative day 0 or 1, followed by 200-m gait training on postoperative day 2. Step-up gait training was initiated only when patients showed no chest pain, severe dyspnea, marked fatigue (Borg score > 13) [6], dizziness, lightheadedness, cyanosis, pallor, or cold extremities; no exercise-related worsening of arrhythmias or conversion to atrial fibrillation; no ischemic electrocardiographic changes during exercise; no excessive blood pressure fluctuations; no exercise-induced heart rate increase >30 bpm; and no oxygen desaturation below 90% [7]. In addition, physical and occupational therapists provide functional training to improve the activities of daily living (ADL) and simultaneously support safe home discharge. Patients who were expected to return home performed ergocycle training with appropriate watts for 20 minutes, followed by resistance training. The resistance training program primarily targeted the major muscle groups and was performed using bodyweight exercises designed to provide a balanced and comprehensive strengthening program. A speech-language-hearing therapist provided swallowing rehabilitation training to patients with difficulty with their oral intake. Physical therapy was provided to all patients, whereas occupational and speech-language therapies were provided on a case-by-case basis.

During hospitalization and after discharge, education on heart failure management was provided orally based on educational materials entitled “Cardiac Rehabilitation for Heart Failure” developed by the Japan Heart Club, with a focus on preventing heart failure onset and exacerbation.

All patients underwent in-hospital rehabilitation based on a standardized institutional protocol. The types of exercise, including early mobilization, gait training, ergocycle exercise, and resistance training, were identical between patients with and without preoperative frailty. Patient demographic and clinical data, such as age, sex, one-year events (death and hospitalization), number of postoperative days, postoperative rehabilitation dose, occupational therapy intervention, speech-language therapy intervention, outpatient rehabilitation, and drug information, were extracted from medical records. All-cause mortality and rehospitalization were also assessed. Rehospitalization was defined as an unplanned hospital admission for any cause. The postoperative rehabilitation dose was defined as the average number of rehabilitation units per day (units/day) provided by physical, occupational, and speech-language-hearing therapists during hospitalization. The rehabilitation dose was calculated by dividing the total number of rehabilitation units during hospitalization by the length of hospital stay (days). One rehabilitation unit was operationally defined as 20 minutes of supervised therapy according to the Japanese National Health Insurance reimbursement system. Exercise modality and intensity were not considered in the calculation of rehabilitation units. The postoperative rehabilitation dose was determined and adjusted daily by therapists according to each patient’s clinical condition and exercise tolerance, following the safety criteria based on current cardiac rehabilitation guidelines.

Decisions were made based on symptoms during exercise, vital signs, electrocardiographic changes, and oxygen saturation. Data on the number of days of early mobilization and frailty were extracted from rehabilitation medical records. Frailty was assessed preoperatively using the Clinical Frailty Scale (CFS), a typical measure used to evaluate frailty. However, it comprises many items and is complicated to calculate, making it difficult to use in clinical situations. However, the CFS score is strongly correlated (r=0.80) with the frailty index, and the risk of mortality and institutionalization increases with worsening CFS scores [8]. The CFS rates frailty with a score of 1 to 9 based on the severity of symptoms and ADL, with scores of 1-4 and 5-9 being indicative of non-frail and frail, respectively. Patients with a CFS score of ≥5 were classified as frail in accordance with previous studies [8]. Trained physical therapists assessed the CFS scores preoperatively.

Furthermore, a short physical performance battery (SPPB) and grip strength tests were used to evaluate physical function. Grip strength was measured twice on each side in the standing posture, and the maximum value of all measurements was used. In patients unable to safely maintain standing posture, handgrip strength was measured in a seated position. The Controlling Nutritional Status score [9] and albumin levels were measured in blood samples collected for nutritional evaluation. Brain natriuretic peptide (BNP) and glomerular filtration rate (GFR) were used to assess heart failure severity and renal function, respectively. Based on the echocardiographic results, we measured the left ventricular ejection fraction (EF) to evaluate left ventricular contractility and the severity of aortic stenosis, and extracted data on the maximum aortic velocity, aortic mean pressure gradient, and aortic valve area. Physical functional assessments, laboratory data, and echocardiographic findings were obtained preoperatively.

In the statistical analysis, characteristics were compared between the preoperative and non-preoperative frailty groups using the Mann-Whitney U or χ2 tests. In addition, Cox proportional hazards models were used to determine independent predictors of one-year mortality and hospitalization. To evaluate the prognostic impact of each frailty domain (physical and nutritional), separate multivariate Cox proportional hazards models were constructed for the SPPB, handgrip strength, and serum albumin level. These variables were not included simultaneously in a single model to avoid potential multicollinearity, as they represent overlapping components of frailty. The CFS was not included in the multivariate models because it is a composite measure that overlaps with its individual components, which could lead to overadjustment and complicate the interpretation of independent effects. Given the limited number of events, we adopted a parsimonious adjustment strategy. All multivariate models were consistently adjusted for age and sex as clinically essential confounders. Kaplan-Meier analysis was performed to evaluate one-year event-free survival, and differences between the low- and high-handgrip strength groups were assessed using the log-rank test. The exploratory cutoff value for handgrip strength was determined using a receiver operating characteristic (ROC) curve. Missing data were minimal, with only one missing value each for handgrip strength. All analyses were performed using R, and p<0.05 was considered statistically significant.

This study was conducted in accordance with the Declaration of Helsinki and the Ethical Guidelines for Medical Research Involving Human Subjects and was approved by the Ethical Review Committee of Ichinomiya Nishi Hospital (approval number: 2025095).

## Results

Patients in the frailty group were significantly older than those in the non-frailty group. The one-year event rate (death and rehospitalization) was significantly higher in patients with preoperative frailty than in those without preoperative frailty (17/43 versus 15/80, p=0.017). The postoperative days were significantly longer in the frailty group than in the non-frailty group. The postoperative rehabilitation doses were 3.6 (2.9-5.0) and 3.2 (2.5-4.2) units/day in the frailty and non-frailty groups, respectively, indicating a significantly higher rehabilitation dose in the frailty group.

Occupational and speech-language therapy interventions were significantly more frequent in the preoperative frailty group than in the non-preoperative frailty group. Regarding physical function, the frailty group showed a significantly lower SPPB of 5.0 (4.0-8.0) compared with 11.0 (9.0-12.0) in the non-frailty group. Handgrip strength was 14.5 (10.7-18.5) kg in the frailty group, and 19.0 (14.0-25.5) kg in the non-frailty group. In terms of nutritional status, the frailty group showed a significantly lower albumin of 3.4 (3.1-3.7) compared with 3.8 (3.3-3.9) in the non-frailty group (Table 1).

**Table 1:** Comparison of patient characteristics Continuous variables were compared using the Mann-Whitney U test, and categorical variables were compared using the χ2 test. a: Mann-Whitney U test, b: χ2 test p-value < 0.05 was considered statistically significant. SPPB: short physical performance battery, CONUT: controlling nutritional status, Alb: albumin, BNP: brain natriuretic peptide, GFR: glomerular filtration rate, EF: ejection fraction, AV peak V: aortic valve area velocity, AV mean PG: aortic valve mean pressure gradient, AVA: aortic valve area, ARB: angiotensin Ⅱ receptor blocker, ACE: angiotensin converting enzyme inhibitor, ARNI: angiotensin receptor neprilysin inhibitor, IQR: interquartile range, BMI: body mass index

Parameters	Preoperative frailty (n=43)	Non-preoperative frailty (n=80)	Test statistics values	p-value
	Median (IQR)			
Age (years)^a^	87.0 (84.0-90.0)	84.0 (82.0-87.0)	U=1016	<0.001
Sex (male/female)^b^	12/31	34/46	χ2=1.96	0.123
BMI (kg/m^2^)^a^	21.5 (19.4-25.0)	22.9 (21.2-24.8)	U=2045	0.064
1-year event (died or rehospitalization/survived)^b^	17/43	15/80	χ2=4.49	0.017
Discharge to home (number (%))^b^	37 (86%)	76 (95%)	χ2=1.92	0.096
Postoperative days (day)^a^	9 (7-12)	8 (5-10)	U=1317	0.031
Postoperative rehabilitation dose (units/day)^a^	3.6 (2.9-5.0)	3.2 (2.5-4.2)	U=1304	0.027
Occupational therapy intervention (yes/no)^b^	20/23	18/62	χ2=6.47	0.008
Speech-language therapy intervention (yes/no)^b^	11/32	5/75	χ2=7.61	0.004
Outpatient rehabilitation (yes/no)^b^	0/43	3/77	χ2=0.45	0.551
Days to start early mobilization (day)^a^	1 (1-1)	1 (1-1)	U=1381	0.164
SPPB^a^	5 (4-8)	11 (9-12)	U=2835	<0.001
Handgrip strength (kg)^a^	14.5 (10.7-18.5)	19.0 (14.0-25.5)	U=2383	<0.001
CONUT (score)^a^	3.0 (2.0-5.0)	2.0 (1.0-3.5)	U=1119	0.093
Alb (g/dL)^a^	3.4 (3.1-3.7)	3.8 (3.3-3.9)	U=2285	0.027
BNP (pg/mL)^a^	232.2 (100.6-611.5)	175.7 (94.7-385.3)	U=1482	0.247
GFR (mL/min/1.73 m^2^)^a^	45.3 (35.3-58.2)	49.0 (37.7-56.3)	U=1796	0.689
EF (%)^a^	59 (48-67)	63 (53-69)	U=1818	0.138
AV peak V (m/s)^a^	4.2 (4.1-4.6)	4.4 (4.1-4.8)	U=1723	0.347
AV mean PG (mmHg)^a^	41.0 (36.3-48.0)	44.0 (40.0-54.8)	U=1877	0.069
AVA (cm^2^)^a^	0.60 (0.52-0.70)	0.63 (0.54-0.81)	U=1754	0.264
ARB/ACE/ARNI (number, (%))^b^	26 (61%)	44 (55%)	χ2=0.15	0.574
Loop diuretics (number, (%))^b^	17 (40%)	26 (33%)	χ2=0.34	0.437

Univariate Cox proportional hazards analysis demonstrated that preoperative frailty (hazard ratio (HR): 2.11, 95% confidence interval (CI): 1.07-4.18, p=0.032) and handgrip strength (HR: 0.95, 95% CI: 0.91-1.00, p=0.034) were significantly associated with one-year post-TAVI outcomes. Age, Sex, SPPB score, albumin level, and ejection fraction were not significantly associated with the outcome (Table 2). In multivariate Cox proportional hazards models adjusted for age and sex, handgrip strength was independently associated with one-year outcomes (HR: 0.93, 95% CI: 0.88-0.98, p=0.006) (Table 3).

**Table 2:** Univariate Cox proportional hazards analysis of one-year post-TAVI outcomes p-value < 0.05 was considered statistically significant. SPPB: short physical performance battery, Alb: albumin, EF: ejection fraction, HR: hazard ratio, CI: confidence interval

Parameters	HR	95% Cl	p-value
Age (years)	1.07	0.99-1.16	0.106
Sex (reference: male)	0.84	0.42-1.68	0.624
Preoperative frailty	2.11	1.07-4.18	0.032
SPPB	0.93	0.84-1.02	0.139
Handgrip strength (kg)	0.95	0.91-1.00	0.034
Alb (g/dL)	0.56	0.30-1.03	0.062
EF (%)	0.98	0.96-1.00	0.158

**Table 3:** Multivariate Cox proportional hazards analysis of one-year post-TAVI outcomes p-value < 0.05 was considered statistically significant. SPPB: short physical performance battery, Alb: albumin, HR: hazard ratio, CI: confidence interval

Model 1: SPPB	HR	95% Cl	p-value
Age (years)	1.07	0.98-1.17	0.134
Sex (reference: male)	0.68	0.33-1.40	0.294
SPPB	0.94	0.86-1.04	0.244
Model 2: Handgrip strength	HR	95% Cl	p-value
Age (years)	1.06	0.97-1.16	0.205
Sex (reference: male)	0.40	0.18-0.87	0.020
Handgrip strength (kg)	0.93	0.88-0.98	0.006
Model 3: Alb	HR	95% Cl	p-value
Age (years)	1.07	0.98-1.18	0.141
Sex (reference: male)	0.69	0.34-1.41	0.311
Alb (g/dL)	0.61	0.33-1.13	0.118

**Figure 1:**
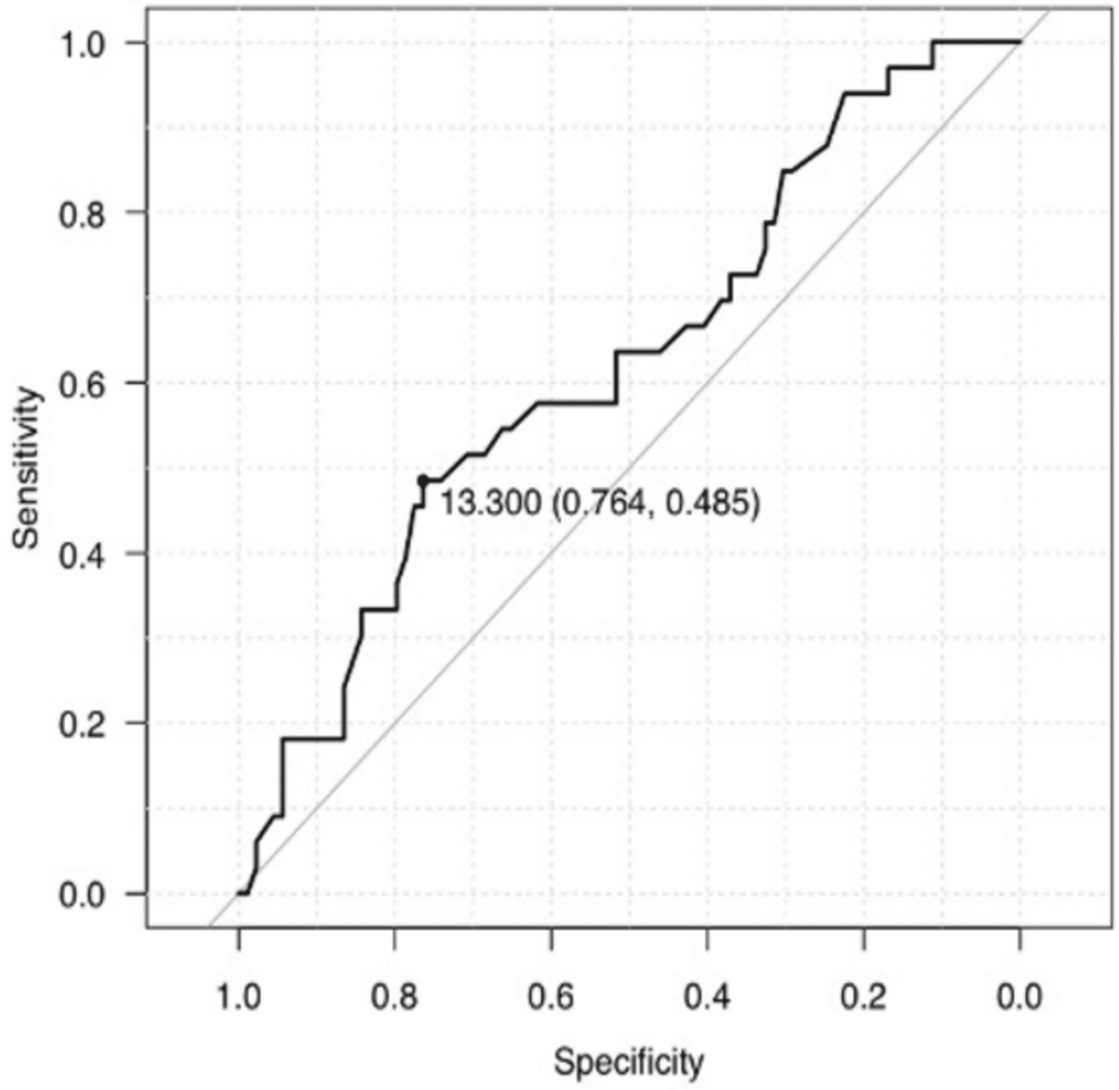
ROC curve of handgrip strength for one-year outcomes after TAVI ROC curve for identifying the exploratory handgrip strength cutoff for the one-year composite outcome A handgrip strength of 13.3 was the exploratory cutoff value (sensitivity: 76.4%, specificity: 51.5%, area under the curve: 0.622). ROC: receiver operating characteristic, TAVI: transcatheter aortic valve implantation

**Figure 2:**
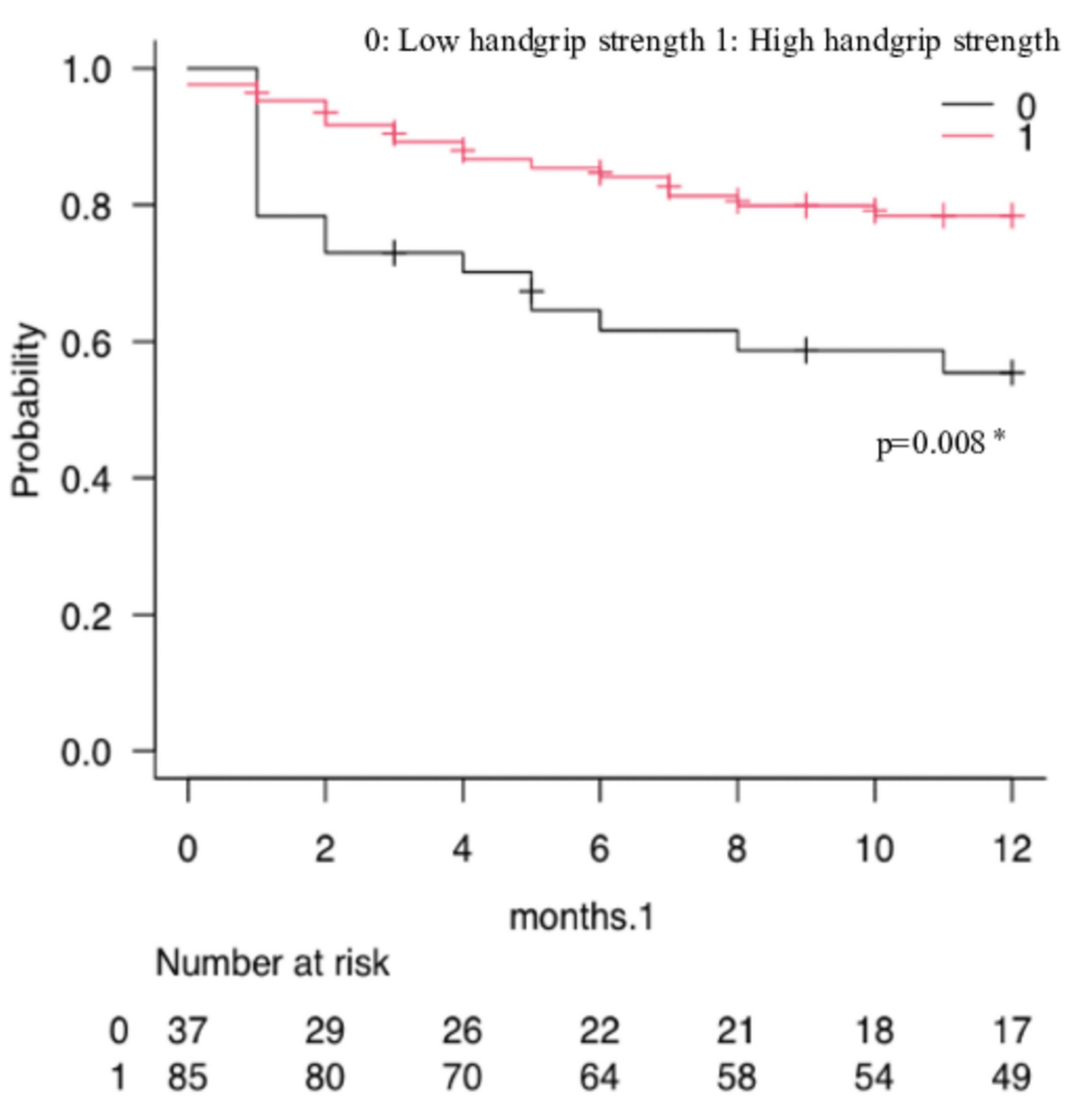
Kaplan-Meier curves for one-year event-free survival according to handgrip strength p-value < 0.05 was considered statistically significant.

ROC curve analysis identified a cutoff value of 13.3 kg for the handgrip strength (Figure 1). Using this cutoff, log-rank testing revealed a significant difference in survival between the high and low handgrip strength groups (Figure 2).

## Discussion

In this study, we investigated the differences in patient characteristics according to the presence or absence of preoperative frailty in patients undergoing TAVI and examined the factors influencing the occurrence of one-year post-procedure outcomes. Patients in the preoperative frailty group showed reduced physical function and nutritional status, along with a longer postoperative hospital stay and a tendency toward a higher number of rehabilitation units per day. In the univariate Cox proportional hazards analysis, preoperative frailty and handgrip strength were significantly associated with the occurrence of one-year events. However, in multivariate Cox proportional hazards analysis, handgrip strength was identified as an independent predictor of one-year events.

In the preoperative frailty group, physical function, assessed using the SPPB and handgrip strength, and albumin levels were impaired. These findings indicate that the participants in this study exhibited multidimensional functional impairments rather than a single functional deficit, consistent with previous reports [1]. Accordingly, patients with frailty required more intensive postoperative rehabilitation, as reflected by the significantly longer length of hospital stay and a higher number of rehabilitation units per day. The rates of home discharge were comparable between the frailty and non-frailty groups.

Given the minimally invasive nature of TAVI and the implementation of standardized early rehabilitation programs, a certain degree of early postoperative functional recovery may have been achieved even in patients with frailty [10]. However, despite this short-term functional recovery, some patients experienced one-year events, including readmission or death, during long-term follow-up. These findings suggest that early postoperative functional recovery alone may not be sufficient to fully explain the long-term prognosis. Lauck et al. reported that early mobilization and exercise therapy after TAVI are safe and feasible and contribute to short-term functional recovery and a reduction in the length of hospital stay [4]. However, the impact of such interventions on long-term outcomes has not been adequately investigated. Taken together, these findings indicate that improvements in short-term outcomes do not necessarily correspond to favorable long-term prognoses.

Therefore, we investigated the factors influencing the occurrence of one-year events as indicators of long-term prognosis. Regression analyses revealed that preoperative frailty was significantly associated with one-year events in the univariate Cox proportional hazards analysis; however, in the multivariate analysis, only handgrip strength remained an independent predictor. These findings suggest that although frailty comprises multiple components, handgrip strength may strongly influence long-term prognosis. Handgrip strength is one of the simplest and most reproducible components of frailty and is a useful prognostic indicator in patients with cardiovascular diseases [11,12].

In contrast, the SPPB was not identified as an independent predictor of long-term prognosis. The association between frailty and SPPB has already been reported in studies targeting individuals aged 65 years and older [13]. However, the SPPB comprehensively assesses overall lower extremity function, including muscle strength, gait performance, and balance ability, and thus reflects global physical function. This multidimensional nature of the SPPB may partly explain the differences observed between SPPB and handgrip strength in predicting long-term prognosis in the present study.

In this study, ROC curve analysis suggested an exploratory handgrip strength cutoff value of 13.3 kg for predicting one-year post-TAVI events. Kaplan-Meier analysis using this cutoff demonstrated a significant difference in event-free survival between patients with high and low handgrip strength, suggesting that handgrip strength may have potential utility for risk stratification in patients undergoing TAVI. Notably, this value was substantially lower than the handgrip strength thresholds proposed by the Asian Working Group for Sarcopenia (AWGS 2019). This finding suggests that the present cutoff may reflect a more advanced state of physical vulnerability, frailty, or reduced physiological reserve rather than sarcopenia alone. Therefore, this value should be interpreted as an exploratory prognostic threshold rather than a diagnostic threshold for sarcopenia.

Handgrip strength is easy to measure and highly reproducible, and is a useful predictor of mortality and cardiovascular events in patients with cardiovascular diseases [14,15]. These findings suggest that handgrip strength may serve as an indicator of physical function reflecting the long-term prognosis of patients after TAVI. Furthermore, because handgrip strength assessment is minimally invasive and can be repeatedly performed, it may be applicable for preoperative risk stratification, postoperative follow-up, and evaluation of the effectiveness of rehabilitation interventions.

Future prospective studies should investigate whether exercise interventions, including those aimed at improving handgrip strength, can contribute to improved long-term prognosis in patients who exhibit low handgrip strength preoperatively or in the early postoperative period. Interventions targeting physical activity and improvements in handgrip strength reduce long-term mortality and cardiovascular risk [16,17]. Given that handgrip strength was identified as an independent predictor of long-term prognosis in this study, it represents a modifiable physical function parameter. Accordingly, improving handgrip strength through exercise therapy may lead to better long-term outcomes in patients undergoing TAVI. Intervention studies with long-term outcomes as the primary endpoints are required to test this hypothesis.

Limitations

This study has several limitations. First, this was a single-center retrospective study, and selection bias could not be excluded. Second, the number of events was relatively small. However, the multivariate models included only three variables (age, sex, and one frailty component), yielding an events-per-variable ratio of approximately 10.7, which was intended to minimize the risk of model overfitting. Third, detailed aspects of rehabilitation content, such as intensity and exercise modality, have not been fully assessed. Fourth, the frailty assessment may have been biased toward the physical domains because cognitive and psychosocial aspects were not comprehensively evaluated. Finally, because this study was conducted in a single-center setting involving an older Japanese population, the external validity of the findings may be limited. These limitations should be considered when interpreting the results. In addition, several variables had missing data due to the retrospective nature of data collection in routine clinical practice, including variability in assessment timing and documentation. This may have introduced a selection bias.

## Conclusions

Preoperative handgrip strength was an independent predictor of one-year post-TAVI outcomes, including all-cause death and rehospitalization. Among the multidimensional components of frailty, handgrip strength demonstrated the strongest association with long-term prognosis after adjustment for age and sex. Given its simplicity and high reproducibility, handgrip strength assessment may serve as a potential tool for preoperative risk stratification and individualized rehabilitation planning in patients undergoing TAVI, although further external validation is required. Furthermore, these findings suggest that targeted rehabilitation strategies focusing on muscle strength and physical function may be associated with improved long-term clinical outcomes in this population.
